# Diagnostic value of an enhanced MRI combined with serum CEA, CA19-9, CA125 and CA72-4 in the liver metastasis of colorectal cancer

**DOI:** 10.1186/s12957-022-02874-x

**Published:** 2022-12-19

**Authors:** Hua-qiang Zhu, Dong-ye Wang, Lin-shen Xu, Jian-le Chen, Er-wei Chu, Cai-jin Zhou

**Affiliations:** 1 Department of Medical Imaging, University of Chinese Academy of Sciences Shenzhen Hospital (Guangming), No. 4253 of Pine White Rd, Guangming District, Shenzhen, 518106 Guangdong Province China; 2 Department of Radiology, Sun Yat-sen Memorial Hospital, Sun Yat-sen University, Guangzhou, 510120 Guangdong Province China

**Keywords:** Colorectal liver metastases, Enhanced magnetic resonance imaging, Carcinoembryonic antigen, Carbohydrate antigen, Consistency

## Abstract

**Objective:**

This paper aims to explore the diagnostic value of enhanced magnetic resonance imaging (MRI) combined with a carcinoembryonic antigen (CEA) and carbohydrate antigen in terms of the liver metastasis of colorectal cancer.

**Methods:**

A total of 167 colorectal cancer patients with liver metastasis and 167 colorectal cancer patients without liver metastasis were selected as the subjects. An automatic electrochemiluminescence analyser was then used to detect the tumour markers CEA, CA19-9, CA125 and CA72-4. The consistency between the MRI examination and clinical pathological examination was also analysed, and the sensitivity, specificity and positive and negative predictive values of various combined detection methods were compared.

**Results:**

The abnormal rates of CEA, CA19-9, CA125 and CA72-4 in the two groups were statistically significant (*P* < 0.05), while the results of the enhanced MRI and clinicopathological examination for liver metastasis in patients with colon cancer were largely consistent (Kappa coefficient = 0.788, *P* < 0.000). However, the two methods were inconsistent. The false positive rate of the enhanced MRI examination was 15.3%, while the false negative rate was 6.0%. The specificity (94.61%), positive predictive value (92.68%) and positive likelihood ratio (12.67%) were the highest for the MRI combined with serial CEA, while the sensitivity (98.80%) and negative predictive value (97.22%) were the highest with the MRI combined with parallel CEA, and this combination returned the lowest negative likelihood ratio (0.03).

**Conclusion:**

The combination of MRI and CEA excludes non-metastatic patients and identifies colorectal liver metastasis cancer patients. Overall, it has a higher diagnostic value.

## Introduction

Colorectal cancer is the third most prevalent cancer globally after lung cancer and adenocarcinoma, with approximately 1.8 million new cases and 900,000 associated deaths reported per year [1]. With advancements in the diagnosis and treatment technology, the 5-year survival rate of patients with early colorectal cancer following surgical resection is 90%; however, the 5-year survival rate of patients with distant metastasis is <20% [2]. The liver is the most affected organ in colorectal cancer, with liver metastasis having the highest incidence and mortality rate among the forms of distant metastasis in patients with colorectal cancer. At present, surgical resection is the best treatment for colorectal liver metastases (CRLM) to ensure long-term survival, but the recurrence rate is high [3]. There are the following two types of CRLM: synchronous and metachronous metastasis. The former refers to liver metastasis occurring upon or before the diagnosis of primary colorectal cancer and accounts for approximately 15–20% of colorectal cancer cases, with the proportion of patients with this form of liver metastasis far lower than that of patients with metachronous metastasis liver metastasis [4]. Early detection of liver metastasis is important in the evaluation of respectability, as it enables the timing of the surgery to be accurately determined or chemotherapy for patients who are not suitable for surgery. In short, early detection improves the timeliness and success rate of the surgical treatment [5].

At present, the most common diagnosis methods for CRLM include computer tomography (CT) and positron emission tomography combined with CT. However, magnetic resonance imaging (MRI) has the advantages of high soft tissue resolution, multi-scan sequence and no radiation hazard. It is an effective technique for detecting small lesions of <1cm and has certain advantages regarding the diagnosis of CRLM [6]. In addition, the antigens, carcinoembryonic antigen (CEA), carbohydrate antigen 19-9 (CA19-9), carbohydrate antigen 125 (CA125) and carbohydrate antigen 724 (CA724), are important tumour markers for the early diagnosis and prognosis of colorectal cancer [7].

However, the above detection methods often involve a certain degree of incorrect and missed diagnoses in their clinical application. In this paper, the diagnostic value of an enhanced MRI combined with CEA, CA19-9, CA125 and CA724 (series and parallel) is evaluated in relation to CRLM in view of identifying a combined detection method with a higher diagnostic value.

## Patients and methods

### Study population

A retrospective study was conducted to select patients with colorectal cancer diagnosed by surgery and pathology in the University of Chinese Academy of Sciences Shenzhen Hospital（Guangming）and Sun Yat-sen Memorial Hospital of Sun Yat-sen University from January 2016 to December 2020 by convenient sampling. The patients with liver metastasis diagnosed by Spiral computer tomography (CT) within 3 months after the first operation were selected as the liver metastasis group (*n*=167). According to the 1:1 matching principle, the patients with non-liver metastasis within 3 months after the first operation were selected as the non-liver metastasis group (*n*=167). All the participants signed an informed consent form, while the study was approved by the ethics committee of the University of Chinese Academy of Sciences Shenzhen Hospital (Guangming). Inclusion and exclusion criteria for study subjects are shown in Table 1.

**Table 1:** Inclusion and exclusion criteria of subjects

Inclusion criteria	Exclusion criteria
Colonoscopic biopsy, diagnostic criteria, clinical and pathological diagnosis of colorectal cancer	Known to have other malignant tumours
No chemotherapy, radiotherapy and hormone therapy before admission	Important organ damage
Complete clinical data	
Willingness to participate voluntarily	

### Research method

#### Serum tumour markers

The main instruments and reagents include a centrifuge instrument, an automatic electrochemiluminescence instrument (Cobas e601), CEA (Abbott GmbH, 2K45-74), CA125 (Abbott GmbH, 7K68-74), CA199 (Abbott GmbH, 2K91-74) and CA72-4 (Roche Diagnostics GmbH, 11776258122). On the second day of admission, 4–5 mL of fasting blood was collected and centrifuged at 3000 rpm for 10 min within 1 h. Serum tumour markers CEA, CA125, CA19-9 and CA72-4 were detected by an automatic electrochemiluminescence analyser. The normal ranges are CEA < 5 ng/mL, CA199 < 37 U/mL, CA199 < 34 U/mL and CA72-4 < 7 U/mL.

#### Enhanced MRI

All patients underwent an MRI examination on the second day of admission. Additionally, all patients fasted for 12 h before examination, and bowel preparation was done. During the examination, the patients were placed in the supine position and scanned using a 1.5T and 3.0T superconducting magnetic resonance scanner (Germany Siemens). The phased array body coil was selected, and a plain scan was performed on cross-sectional T1WI (parameters: TR 549 ms, TE8 ms, layer thickness 5 mm, layer spacing 1 mm, matrix 330 × 322; field of view 300 mm × 360 mm) and T2WI (parameters: TR 3400 ms, TE 80 ms, slice thickness 5 mm, layer spacing 1 mm, matrix 272 × 291; vision 300 mm × 360 mm). Moreover, t2WI was performed on coronal and sagittal planes (parameters: TR 3500 ms, TE 80 ms, slice thickness 5 mm, slice spacing 1 mm, matrix 320 × 260, vision 250 mm × 250 mm). After an intravenous injection of 0.1 mmoL/kg Gd-DTPA, the patients were scanned using a fat-suppressed fast gradient echo sequence for cross-sectional (slice thickness 5 mm, layer spacing 0.5 mm) and coronal (slice thickness 7 mm, layer spacing 3.5 mm) dynamic enhanced scans. The diffusion-weighted imaging scan parameters are application of plane echo imaging sequence, TR 2370 ms, TE 74 ms, slice thickness 4 mm, layer spacing 0.4 mm and visual field 280 mm × 280 mm.

### Observed indexes

The consistency of the MRI and clinical pathological examinations was assessed, and the sensitivity, specificity and positive and negative predictive values of various combined detection methods were compared. The sensitivity test entailed the probability of being tested positive among individuals with the disease (positive), as assessed according to the gold standard, while the specificity test entailed the probability of returning a negative result according to the gold standard assessment of no disease (negative). Meanwhile, a positive predictive value refers to the proportion of truly ‘diseased’ cases (true positives) among all the positive cases detected by the screening test, while a negative predictive value refers to the proportion of subjects with a negative test result in relation to all truly negative subjects.

### Statistical analysis

The data was analysed using SPSS21.0 software. The quantitative data were described in terms of mean ± standard deviation (*X* ± *S*), while the qualitative data were described in terms of *n* and the rate or composition ratio (*n*%). The difference between the groups was assessed using the 2-test, while the consistency was analysed using the Kappa test. *P* < 0.05 was regarded as statistically significant.

## Results

### Patient characteristics

There were 107 males and 60 females in the liver metastasis group, with an average age of 57.44 ± 13.55, while there were 112 males and 55 females, with an average age of 53.49 ± 14.49, in the non-liver metastasis group. There was no significant difference in terms of age and gender between the two groups. There were 14 cases of transverse colon cancer, nine cases of descending colon cancer, 32 cases of ascending colon cancer, 54 cases of sigmoid colon cancer and 58 cases of rectal cancer in the liver metastasis group, while there were 10 cases of transverse colon cancer, 15 cases of descending colon cancer, 39 cases of ascending colon cancer, 56 cases of sigmoid colon cancer and 47 cases of rectal cancer in the non-liver metastasis group. There was no significant difference in terms of the primary lesion composition between the two groups. The abnormal rates of the tumour markers in the liver metastasis group were higher than those in the non-liver metastasis group (Table 2).

**Table 2:** Patient baseline demographics and clinical characteristics

		Non-liver metastasis group	Liver metastasis group	*χ*^*2*^*/t*	*P*
Sex^a^	Male	112 (67.07%)	107 (64.07%)	0.332	0.565
	Female	55 (32.93%)	60 (35.93%)		
Age^a^ (years)		53.49 ± 14.49	57.44 ± 13.55	1.568	0.093
Primary lesions^a^	Transverse colon cancer	10 (2.99%)	14 (8.38%)	4.498	0.480
	Lower colon cancer	15 (4, 49%)	9 (5.39%)		
	Colon cancer	39 (11.68%)	32 (19.17%)		
	sigmoid colon cancer	56 (16.77%)	54 (32.33%)		
	Rectal cancer	47 (14.07%)	58 (34.73%)		
Tumour marker^b^	Abnormal CEA	114 (62.26%)	147 (88.02%)	19.090	0.001*
	Abnormal CA125	99 (59.28%)	128 (76.65%)	11.565	0.001*
	Abnormal CA19-9	117 (70.06%)	142 (85.03%)	10.746	0.001*

*The difference was statistically significant

^a^*N* (constituent ratio)

^b^*N* (rate)

### Enhanced MRI comparison

The consistency between the MRI examination and clinical pathological examination was analysed, with the results for liver metastasis in patients with colon cancer consistent between the two methods (Kappa coefficient = 0.788, *P* < 0.000). However, the two methods demonstrated some inconsistencies. The false positive rate of the enhanced MRI procedure was 15.3%, while the false negative rate was 6.0% (Table 3).

**Table 3:** The consistency between MRI examination and clinical pathological examination

		Clinical pathological		Kappa	*P*
		Metastasis	Non-metastasis		
MRI	Metastasis	157 (94.0%)	29 (15.3%)	0.788	<0.001
	Non-metastasis	10 (6.0%)	138 (4.7%)		

### Enhanced MRI combined with tumour markers

Given that the abnormal rates of the tumour markers differed between the liver metastasis group and the non-liver metastasis group, the MRI and tumour markers were combined to examine whether liver metastasis occurs in patients with colon cancer. MRI with serial CEA examination showed the highest agreement with clinicopathological examination, and the results of liver metastasis were consistent between the two colon cancer patients (Kappa coefficient=0.629, *P* < 0.001). The concordance of the remaining MRI combined with tumour markers CEA, CA19-9, CA125 and CA72-4 with MRI in parallel and tandem examinations is shown in Table 4.

**Table 4:** Results of enhanced MRI combined with tumour markers

			Clinical pathological			
			Metastasis	Non-metastasis	Kappa	*P*
CEA	Parallel	Metastasis	165 (98.80%)	97 (58.08%)	0.318	<0.001
		Non-metastasis	2 (0.20%)	70 (41.92%)		
	Serial	Metastasis	114 (68.26%)	9 (5.39%)	0.629	<0.001
		Non-metastasis	53 (31.74%)	158 (94.61%)		
CA19-9	Parallel	Metastasis	152 (91.02%)	101 (59.88%)	0.305	<0.001
		Non-metastasis	15 (8.98%)	66 (40.12%)		
	Serial	Metastasis	80 (47.90%)	13 (7.78%)	0.401	<0.001
		Non-metastasis	87 (52.10%)	154 (92.22%)		
CA125	Parallel	Metastasis	148 (88.62%)	110 (65.87%)	0.228	<0.001
		Non-metastasis	19 (11.38%)	57 (34.13%)		
	Serial	Metastasis	46 (27.54%)	21 (12.57%)	0.150	0.001
		Non-metastasis	121 (72.46%)	146 (87.43%)		
CA72-4	Parallel	Metastasis	156 (93.41%)	136 (81.44%)	0.162	<0.001
		Non-metastasis	11 (6.59%)	41 (18.56%)		
	Serial	Metastasis	100 (59.88%)	14 (8.38%)	0.515	<0.001
		Non-metastasis	67 (40.12%)	153 (91.62%)		

Here, MRI with parallel CEA had the highest sensitivity (98.80%), MRI with serial CEA had the highest specificity (94.61%) and the highest positive predictive value (92.68%), MRI with parallel CEA had the highest negative predictive value (97.22%), MRI with serial CEA had the highest positive likelihood ratio (12.67%) and MRI with parallel CEA had the lowest negative likelihood ratio (0.03). MRI with serial CEA had the highest accuracy (81.43%). In sum, the specificity, positive predictive value and positive likelihood ratio were the highest for the MRI with the serial CEA, and the sensitivity and the negative predictive value were the highest for the MRI with the parallel CEA. This combination had the lowest negative likelihood ratio (Table 5).

**Table 5:** Various combined diagnostic indicators

	Sensitivity (%)	Specificity (%)	Positive predictive value (%)	Negative predictive value (%)	Positive likelihood ratio (%)	Negative likelihood ratio (%)	Accuracy
MRI serial CEA	68.26	94.61	92.68	74.88	12.67	0.34	81.43
MRI parallel CEA	98.80	41.92	62.98	97.22	1.70	0.03	70.35
MRI serial CA19-9	47.90	92.22	86.02	63.90	5.18	0.56	70.01
MRI parallel CA19-9	91.01	39.52	60.08	81.48	1.50	0.23	65.27
MRI serial CA125	27.54	87.43	68.66	54.68	2.19	0.83	57.49
MRI parallel CA125	88.62	34.13	57.36	75.00	1.35	0.33	61.38
MRI serial CA72-4	59.88	91.62	87.72	69.55	7.14	0.44	75.57
MRI parallel CA72-4	93.41	24.55	53.42	78.85	1.15	0.27	58.98

## Discussion

The early and accurate detection of CRLM is of great significance for the treatment and prognosis of colorectal cancer patients. However, due to the lack of specific symptoms and the limited detection methods, CRLM often entails a missed diagnosis, with the disease entering the later stages when obvious symptoms appear [8, 9]. Previous studies have demonstrated that upon diagnosis, approximately 80% of CRLM patients have lost the opportunity to eliminate the metastasis via surgery and are often left with a survival rate of only a few months. However, the 5-year survival rate of CRLM patients can reach up to 40% if the liver metastasis is completely eliminated through surgery [10]. At present, the common CRLM imaging detection methods include CT, ultrasound and enhanced MRI examinations. An enhanced MRI has the advantages of presenting an enhancement of the lesions, being more sensitive to small lesions and being able to detect liver metastasis early. The technique also evaluates the residual volume of the liver, which provides a reference for surgical planning and improves the success rate of the surgery [11].

CEA is one of the first and most widely used tumour markers. It is a specific acidic glycoprotein of a human embryonic antigen. Studies have found that the CEA expression level and distant metastasis of colorectal cancer have a certain correlation when CEA > 15ng/mL of colorectal cancer patients with postoperative distant metastasis probability is high [4], but the false positive and false negative situation is more. CA19-9 is a related antigen secreted by digestive system tumour cells, which is highly expressed in gastric, colon, rectal and pancreatic cancer tissues. Studies have shown that CEA, CA19-9 and CA125 are significant in predicting liver metastasis of colorectal cancer. Among them, CEA is significant in predicting the T stage, CA19-9 is significant in predicting the T and N stages and CA125 is significant in predicting the degree of differentiation of the primary tumour.

In the present research, the results of the enhanced MRI examination and clinicopathological examination for liver metastasis in patients with colon cancer were found to be consistent (Kappa coefficient = 0.788, *P* < 0.000). However, the two methods demonstrated some inconsistencies. The sensitivity and specificity of the enhanced MRI examination were 94.0% and 84.7%, respectively, which indicated that given the 1.8 million new cases of colon cancer reported each year, many people could be misdiagnosed or have a missed diagnosis when using an MRI examination alone. To improve the outcome for CRLM patients, other detection methods could be combined with an MRI examination to improve the sensitivity and/or specificity.

In the occurrence and progression of colorectal cancer, the tumour markers will change due to the expression and accumulation of multiple genes. Serological tumour markers have the advantages of a fast diagnosis and small trauma diagnosis. A study on the relationship between serological indexes and pathological parameters in 279 patients with colorectal cancer found that the sensitivity of CEA, CA19-9, CA72-4 and CA125 to colon cancer was in the top five of the serum blood indexes studied and was associated with pathological tumour lymph node metastasis and vascular invasion. This indicates that it may be related to the liver metastasis of colon cancer. However, there is no consensus on the diagnostic criteria for CRLM patients in terms of the above indicators [12, 13].

In the present study, the highest specificity (94.61%), positive predictive value (92.68%) and positive likelihood ratio (12.67%) were obtained with an MRI examination combined with a serial CEA. However, this method could exclude colon cancer patients without liver metastasis. The combination of an MRI and parallel CEA had the highest sensitivity (98.80%) and negative predictive value (97.22%) but the lowest negative likelihood ratio (0.03), which means that this combination could improve the referral rate of CRLM patients.

CEA is produced by the digestive tract. The expression level of CEA is extremely low in healthy individuals but is significantly increased when a digestive tract tumour occurs [14]. As such, CEA is a relatively broad-spectrum tumour marker, with its expression increased in liver, colon and gastric cancer patients. The antigen can easily appear on the surface of cancer cells; is captured by Kupffer cell-specific receptors in the liver; can induce the expression of IL-1α, IL-1β and other cytokines, as well as specific cancer-cell adhesion factors; and can promote the retention of cancer cells in the capillary network and liver metastasis [15]. The CEA expression level in the serum of CRLM patients will be significantly increased, and previous studies have found that CEA levels are associated with the prognosis of patients with colon cancer metastasis [16]. Since the 5-year survival rate after early surgical resection of CRLM lesions is generally greatly improved, the recommendation is to use the method of enhanced MRI combined with CEA to provide a reference for the early diagnosis of liver metastasis in patients with colon cancer and evaluate the surgical feasibility of patients in a timely manner.

As this study only included the patients from one hospital as the research subject, some admission rate bias could have been introduced. Furthermore, no other imaging detection method was designed in the experimental scheme, which was not conducive to a more objective evaluation of the diagnostic value of an MRI examination combined with serological indexes. Therefore, multi-centre clinical trials must be carried out in the future, with a variety of methods used as controls to further evaluate the diagnostic value of an MRI examination combined with serological indicators for CRLM.

## Conclusion

In summary, although the consistency between the CRLM results of an enhanced MRI examination and clinical pathological detection is strong, the sensitivity and specificity must be improved. A combination of MRI and the serological index, CEA, was found to improve the specificity and sensitivity, and such a method is worthy of promotion in China.

## Data Availability

All data generated or analysed during this study are included in this published article.
