# Correction: Diagnostic value of an enhanced MRI combined with serum CEA, CA19-9, CA125 and CA72-4 in the liver metastasis of colorectal cancer

**DOI:** 10.1186/s12957-023-02980-4

**Published:** 2023-03-13

**Authors:** Hua-qiang Zhu, Dong-ye Wang, Lin-shen Xu, Jian-le Chen, Er-wei Chu, Cai-jin Zhou

**Affiliations:** 1 Department of Medical Imaging, University of Chinese Academy of Sciences, Shenzhen Hospital (Guangming), No. 4253 of Pine White Rd, Guangming District, Shenzhen, 518106 Guangdong Province China; 2 Department of Radiology, Sun Yat-sen Memorial Hospital, Sun Yat-sen University, Guangzhou, 510120 Guangdong Province China

**Correction: World J Surg Onc 20, 401 (2022)**

**https://doi.org/10.1186/s12957-022-02874-x**

Following publication of the original article [1], the authors identified an error in the maintext of the article. Please see below for the changes in bold.

**Table:** 

From	To
A retrospective study was conducted to select patients with colorectal cancer diagnosed by surgery and pathology in the **University of Chinese Academy of Sciences Shenzhen Hospital (Guangming)** from January 2016 to December 2020 by convenient sampling.	A retrospective study was conducted to select patients with colorectal cancer diagnosed by surgery and pathology in the **University of Chinese Academy of Sciences Shenzhen Hospital (Guangming) and Sun Yat-sen Memorial Hospital of Sun Yat-sen University** from January 2016 to December 2020 by convenient sampling.
The patients with liver metastasis diagnosed by **positron emission tomography (PET CT)** within 3 months after the frst operation were selected as the liver metastasis group (n=167).	The patients with liver metastasis diagnosed by **Spiral computer tomography ( CT )** within 3 months after the frst operation were selected as the liver metastasis group (n=167).
During the examination, the patients were placed in the supine position and scanned using a **3.0T super conducting magnetic resonance scanner (Germany Siemens).**	During the examination, the patients were placed in the supine position and scanned using a **1.5T and 3.0T superconducting magnetic resonance scanner (Germany Siemens).**

The original article has been updated.
